# LPM3770277, a Potent Novel CDK4/6 Degrader, Exerts Antitumor Effect Against Triple-Negative Breast Cancer

**DOI:** 10.3389/fphar.2022.853993

**Published:** 2022-04-11

**Authors:** Jiahao Qiu, Xinfa Bai, Wenjing Zhang, Mingxu Ma, Wenyan Wang, Ye Liang, Hongbo Wang, Jingwei Tian, Pengfei Yu

**Affiliations:** ^1^ Key Laboratory of Molecular Pharmacology and Drug Evaluation (Yantai University), School of Pharmacy, Ministry of Education, Collaborative Innovation Center of Advanced Drug Delivery System and Biotech Drugs in Universities of Shandong, Yantai University, Yantai, China; ^2^ Luye Pharma Group, Yantai, China; ^3^ School of Pharmacy, Binzhou Medical University, Yantai, China

**Keywords:** triple-negative breast cancer, CDK4/6 degrader, hydrophobic tagging, proteasome, lysosome-promoted autophagy

## Abstract

Triple negative breast cancer (TNBC) is a subtype of breast cancer with significant malignancy and poor prognosis but effective treatments are limited. Given the critical role of CDK4/6 in cell cycle and the apparent success of CDK4/6 inhibitors against certain cancer, this study attempted to utilize hydrophobic tagging technology to develop a CDK4/6 degrader against TNBC. We based on the chemical structure of the major metabolite of a clinically approved CDK4/6 inhibitor, abemaciclib, to synthesize three compounds and evaluated their *in vitro* cytotoxicity. LPM3770277 stood out as the most promising compound which was further confirmed by a series of binding and CDK4/6 degradation studies. LPM3770277 was able to bind to CDK4/6, and time-dependently and dose-dependently increased CDK4/6 protein degradation. Mechanistic study revealed that LPM3770277 exerted its CDK4/6 degradation effect *via* two machineries: proteasome and lysosome-promoted autophagy. Using *in vivo* TNBC xenograft cancer model, we found that LPM3770277 demonstrated superior anti-tumor efficacy and safety as compared to abemaciclib, although both compounds exerted similar effects on cell cycle arrest. In conclusion, this study for the first time developed and characterized a CDK4/6 degrader against TNBC using hydrophobic tags, which strongly suggests the viability of hydrophobic tags as a strategy to develop potential treatments against TNBC.

## Introduction

Within the spectrum of breast cancer, triple negative breast cancer (TNBC) is known as a type of breast cancer that lacks the expression of estrogen receptor, progesterone receptor and human epidermal growth factor receptor 2 (Ryu et al., 2011). Clinically, TNBC is considered an aggressive subtype of breast cancer with a poor prognosis and more prone to early recurrence. Identification of effective therapeutic targets and development of new treatments of TNBC are in dire need.

Cyclin-dependent kinases 4 and 6 (CDK4/6) regulate the G1-S cell cycle transition by phosphorylating the tumor suppressor retinoblastoma (Rb), thereby triggering gene expression programs that promote S phase entry (O’Leary et al., 2016). As such, CDK4/6 are attractive targets for cancer therapy and several CDK4/6 inhibitors including palbociclib, ribociclib and abemaciclib have been approved for treating patients with advanced or metastatic breast cancer, which demonstrate good clinical results (Laderian and Fojo, 2017). Unfortunately, TNBC is not sensitive to CDK4/6 inhibitors (Lehmann et al., 2011; Asghar et al., 2017). However, despite the fact that TNBC may not be particularly sensitive to CDK4/6 inhibitors, CDK4/6 protein is indispensable for the proliferation of Rb wild-type triple-negative breast cancer (Fassl et al., 2020). Therefore, exploring alternative approaches to perturb CDK4/6 function still has its merit as a possible therapeutic strategy to treat TNBC.

Small-molecule hydrophobic tagging is an effective approach to degrade rather than inhibit target proteins (Gustafson et al., 2015). The hydrophobic tag on a small molecule is embedded on the surface of the target protein, which is mistaken as a partially misfolded protein by the cell’s protein repair mechanism which will then be degraded by the proteasomes (Lai and Crews, 2017; Wang et al., 2020). This technology has been proven as a valid drug discovery approach. For example, the selective estrogen receptor degrader fulvestrant is the first-in-class drug approved for the treatment of hormone receptor-positive, HER2-netagive, advanced stage breast cancer (McDonnell et al., 2015).

In this study, we designed a novel hydrophobic tagging compound LPM3770277 aiming to degrade CDK4/6 protein in TNBC. We found that as compared to CDK4/6 inhibitors, LPM3770277 could degrade CDK4/6 protein through the proteasome system and lysosomal system, which led to more efficacious anti-TNBC growth activity and had less adverse effects. These results suggest the possibility of targeting CDK4/6 degradation for the treatment of TNBC.

## Materials and Methods

### Antibodies and Chemicals

The following antibodies were used in the study: CDK4 (Abcam, Cambridge, United Kingdom, 1:1,000), CDK6 (Abcam, Cambridge, United Kingdom, 1:50,000), Rb (Abcam, Cambridge, United Kingdom, 1:2000), phospho-Rb S780 (Abcam, Cambridge, United Kingdom, 1:1,000), LC3B (Cell Signaling Technology, Danvers, MA, United States, 1:1,000), P62 (Cell Signaling Technology, Danvers, MA, United States, 1:1,000), LAMP2 (Cell Signaling Technology, Danvers, MA, United States, 1:1,000), β-actin (Beyotime, Shanghai, China, 1:1,000). Abemaciclib and Abemaciclib metabolite M2 were purchased from MedChemExpress (Monmouth Junction, NJ, United States, LY-2835219, LSN2839567). Compounds LPM3770277, LPM3770278, and LPM3770304 were synthesized by Shandong Luye Pharmaceutical Corporation (Yantai, China).

### Animals

Adult female Balb/c nude mice (initial weight 18–22 g) were purchased from Beijing Vital River Laboratory Animal Technology Corporation (Beijing, China). Mice were group housed on a 12/12-h light/dark cycle with free access to water and food. The study was approved by the Experimental Animal Ethics Committee of Yantai University, China, and complied with the 2011 Guide for the Care and Use of Laboratory Animals (Institute of Laboratory Animal Resources on Life Sciences, National Research Council, National Academy of Sciences, Washington DC, United States).

### Cell Culture

The HCC1806 breast cancer cell line was purchased from ATCC (Manassas, VA, United States). The mesenchymal triple-negative breast cancer cell line SUM159 was purchased from Beina Bio (Changsha, China). The cells were cultured in opti-1640 media supplemented with 10% fetal bovine serum (FBS) and 1% penicillin-strepto-mycin, and incubated at 37°C in a humidified atmosphere containing 5% CO_2_. All cells were harvested during the exponential growth phase for assays.

### Cell Proliferation Assay

Celltiter-Glo Luminescent Cell Viability Assay (Promega USA) was used to detect cell viability according to manufacturer’s instructions. This assay is a homogeneous method of determining the number of viable cells in culture based on quantitation of the ATP present, an indicator of metabolically active cells. Briefly, cells were digested and paved in black 96-well plates with 3,000 cells/100 µl cell suspension solution, then different concentrations of treatment drugs were added the next day, and placed in an incubator for 72 h. Then, add 100 µl Celltiter-Glo solution, mix well for 2 min, incubate at room temperature for 10 min, detect the luminous intensity of each well with spectrophotometer (BioTek, Winooski, VT, United States). Repeat the experiment three times to calculate the cell survival rate.

### Western Blot

Cells were collected after incubation and lysates were prepared to detect the target proteins as previously described (Ahmed et al., 2019). Briefly, cells were exposed to the test compounds at the indicated concentrations and then collected and lysed in RIPA (Radio Immuno-precipitation Assay) buffer. The total cellular protein extract was electrophoresed on 8% SDS-polyacrylamide gels and then transferred to a PVDF membrane (Millipore, Burlington, MA, United States). The membranes were incubated overnight with primary antibodies, followed by incubation with secondary antibodies. The membrane was developed and then visualized by Image Quant LAS4000 (GE, United States).

### Fluorescence Polarization Binding Assay

The assay was performed according to manufacturer’s instructions (BPS Bioscience, San Diego, CA, United States). Briefly, using a black 96-well plate, add the following substances to each well: 6 µl 5 × Kinase assay buffer 1, 1 µl ATP (500 µM), 5 µl 10 × CDK4 substrate peptide, 13 µl deionized water, and 5 µl Inhibitor Buffer was added to the Positive Control group (no inhibitor), and 20 µl CDK4/CyclinD3 solution (10 ng/µl). Add 5 µl of compound solutions (1.5625, 3.125, 6.25, 12.5, 25, 50, 100 and 500 nM of abemaciclib or the same concentrations of LPM3770277) and 20 µl CDK4/CyclinD3 (10 ng/µl) to the Test Inhibitor group. Add 5 µl Inhibitor Buffer (no inhibitor) and 20 µl 1x Kinase buffer one to the Blank group. Add 50 µl to each well after incubating with Kinase Glo®Max reagent for 60 min at 30 C. Cover the plate with aluminum foil and incubate at room temperature for 10–15 min. Use a microplate reader to measure the luminous intensity of each well. Subtract the “blank group” value from all readings.

### Cell Cycle Detection

Cells were plated at 150,000 cells/ml overnight in six-well plates. Abemaciclib and LPM3770277 were added in a dose-dependent fashion and then cells were incubated for 48 h. The cells were then fixed with 70% ethanol overnight at −20°C. The cells were centrifuged, and ethanol was removed. The cells were washed twice with 1 × PBS following staining with DAPI (4′,6′diamino-2-phenylindole) and analyzed using BD LSR II (Becton Dickenson, Franklin Lakes, NJ, United States) with a blue filter. Experiments were performed in triplicate.

### Tumor Xenograft Experiments

Nude mice (6–8 weeks old, BALB/c, female) were used to develop the ectopic xenograft tumor model following our published protocol (Ma et al., 2018). Briefly, HCC1806 or SUM159 cells (5 × 10^6^) were implanted into the back of the mice by subcutaneous injection. When the tumor mass reached 100 mm^3^, the mice were randomly divided into three groups (*n* = 8 per group): control group, Abemaciclib group (50 mg/kg) and LPM3770277 group (57.9 mg/kg). We chose Sodium carboxymethyl cellulose (SCMC) solution as the solventfor LPM3770277 with Abemaciclib, and sonicated for 45 min to make it into a suspension. The control group was given 0.2 ml of normal saline per day, Abemaciclib group and LPM3770277 group were given 0.2 ml of drug solutions per day. The tumor growth was measured every third day during the treatment. At the end of the treatment, mice were sacrificed and the tumors were removed and weighed.

### Blood Sampling and Test

Nude mice (18–20 g) were randomly divided into three groups (*n* = 6 per group) and they were orally administered with 0.2 ml of normal saline, Abemaciclib (50 mg/kg), or LPM3770277 (57.9 mg/kg) after administration for 21 days, then blood was taken for analysis.

### Determination of the Drug Concentrations in Lysosomes

Lysosome Enrichment Kit (Thermo Fisher Scientific, Waltham, MA, United States) was used to isolate and enrich lysosomes according to manufacturer’s instructions. Briefly, cells were digested and plated and drugs (Abemaciclib or LPM3770277 (1 μM)) were added. 24 h later, centrifuge the harvested cell to obtain 50–200 mg cell particles. Add 800 µl of Lysosomal Enrichment Reagent A, spin for 5 s and incubate on ice for 1 min. Sonicate the cell suspension on ice. Then add 800 µl of Lysosomal Enrichment Reagent B and centrifuge the tube at 500 × g for 10 min at 4°C. Collect the supernatant in a new tube and store on ice. In the centrifuge tube, add the OptiPrep gradient solution and mix the prepared cell extract with the OptiPrep cell separation medium to obtain a final concentration of 15% OptiPrep medium. At 4°C, the sample was ultracentrifuged at a speed of 145,000 × g for 2 h. After centrifugation, several band gradients would form. Remove the top 2 ml of the gradient (lysosomal layer) and store on ice. The separated lysosome fraction was mixed with 2–3 volumes of PBS to reduce the concentration of OptiPrep medium. Vortex gently to mix the sample. Transfer the sample to a microcentrifuge tube and centrifuge at 18,000 × g for 30 min at 4°C. Remove the supernatant and place the lysosome pellet on ice. Add 1 ml of gradient dilution buffer to wash the particles, and centrifuge at 18,000 × g for 30 min at 4°C. The supernatant was removed, and the lysosomal particles were stored on ice. After adding ultrapure water, HPLC was used to detect the drug concentration in the lysosomes.

### Immunohistochemistry

At the end of the xenograft tumor experiment, the collected tumor samples were fixed in 4% paraformaldehyde solution, dehydrated, and embedded in paraffin. As described earlier (Sun et al., 2018), CDK4 and CDK6 were immunohistochemically stained on paraffin-embedded sections with a thickness of 4 μm. Briefly, the sections were blocked with 3% normal goat serum and incubated with cleaved CDK4 (1:200) and CDK6 (1:200) antibodies at 4°C overnight, and then incubated with the corresponding biotinylated secondary antibodies. The sections were developed with DAB (3,3′-diaminobenzidine) and counterstained with hematoxylin. The Vectra automated quantitative imaging system (Perkinlemer, Waltham, MA, United States) was used to examine the slides under a high-power microscope (200×).

### Statistical Analyses

GraphPad Prism 8.0 Software (GraphPad Software, San Diego, CA, United States) was used for one-way ANOVA and two-way ANOVA analysis. Data were expressed as mean ± SD. *p* < 0.05 was considered statistically significant.

## Results

### Compound Design and Screening

**FIGURE 1:**
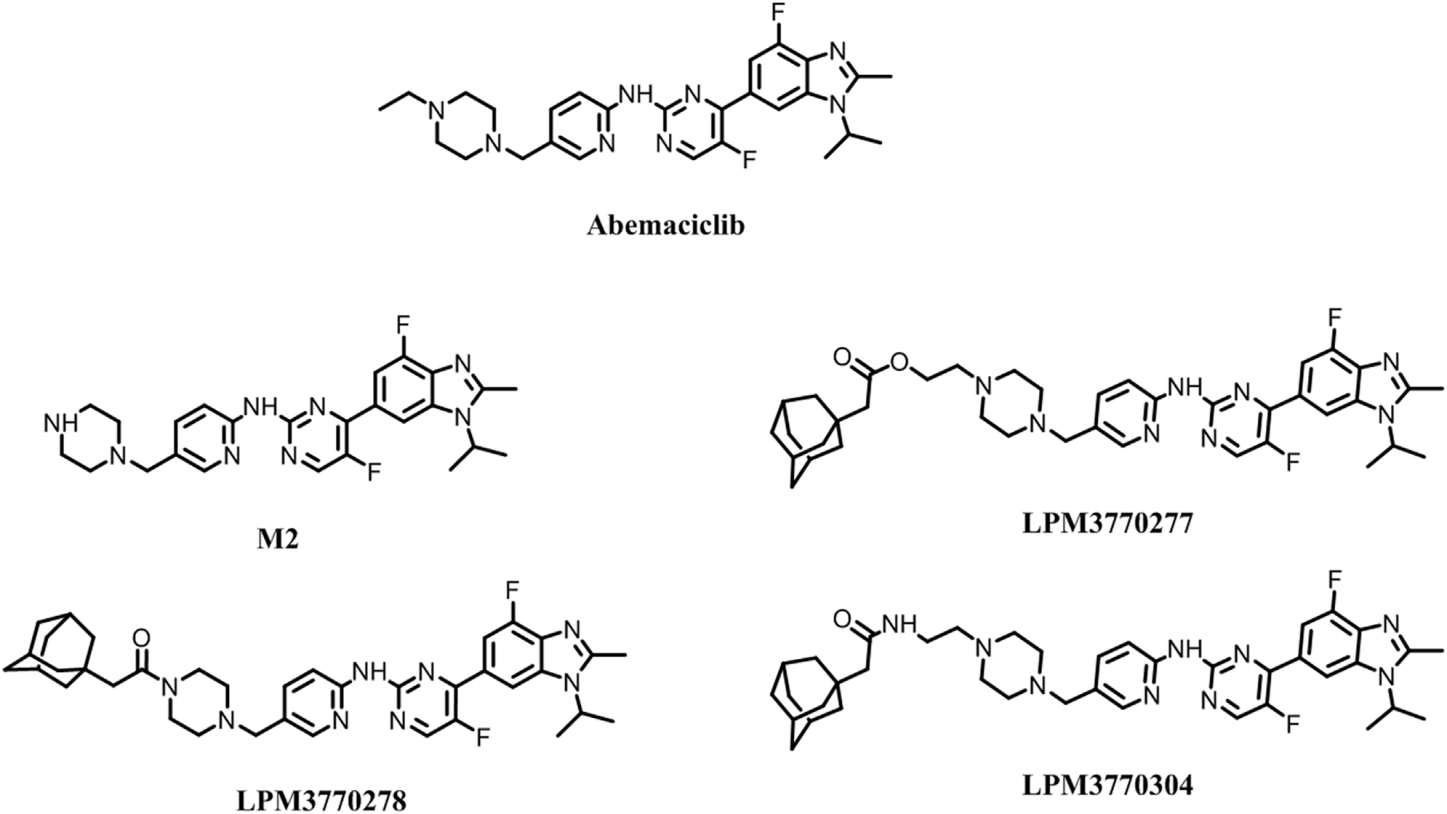
Chemical structures of the compounds.

**FIGURE 2:**
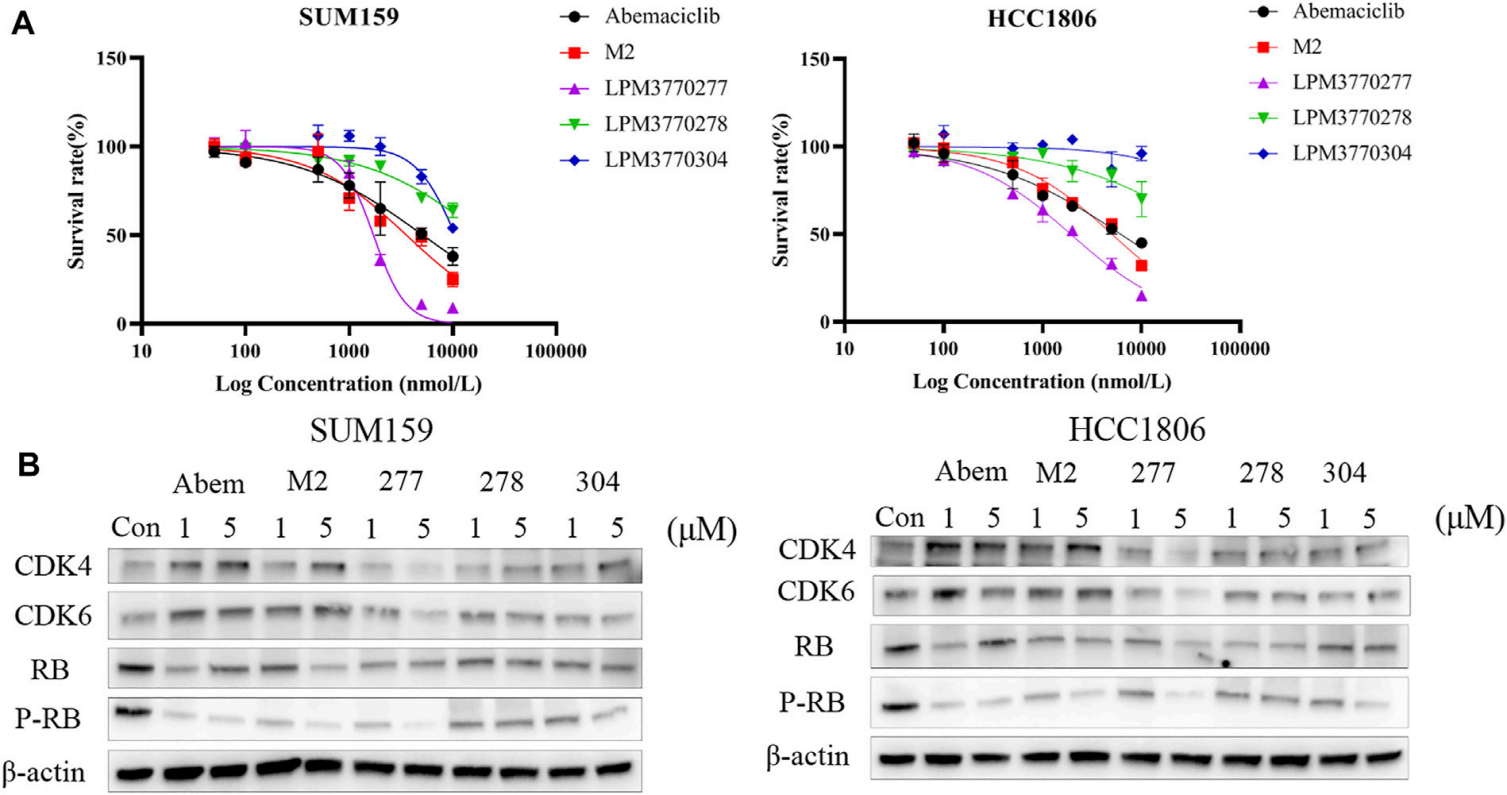
Initial screening of the compounds. **(A)**: The effects of five compounds on the survival rates of two Rb wild-type triple-negative breast cancers cells HCC1806 and SUM159. **(B)**: Effects of five compounds on CDK4, CDK6, Rb, P-Rb of HCC1806, and SUM159 cells. Abem: Abemacialib, 277:LPM3770277, 278:LPM3770278, 304:LPM3770304.

Based on the chemical structure of the active metabolite, M2, of the existing CDK4/6 inhibitor abemaciclib, we have developed three new compounds as shown in Figure 1, and screened their activities as shown in Figure 2A: in the cytotoxicity experiments using TNBC cell lines HCC1806 and SUM159, we found that compound LPM3770277 demonstrated the best efficacy and was more toxic to these two types of cancer cells than abemaciclib and its active metabolite M2. Then we tested the effects of these compounds on CDK4/6 protein as shown in Figure 2B: abemaciclib and its active metabolite M2 significantly increased the expression of CDK4/6 in cells, which were consistent with their effects as inhibitors of CDK4/6. In contrast, LPM3770277 markedly degraded CDK4/6 protein at a concentration of 2 μM, which suggested that in RB wild-type TNBC, LPM3770277 was an effective CDK4/6 protein degrader.‬‬‬‬‬‬‬‬‬‬‬‬‬‬‬‬‬‬‬

### Time Course and Dose Dependency of LPM3770277 on CDK4/6 Protein Expression

**FIGURE 3:**
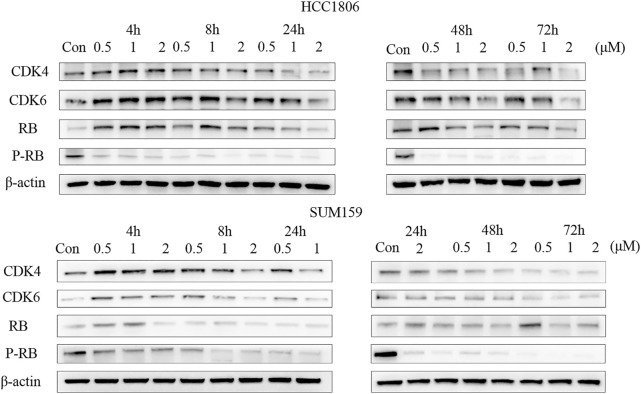
The effect of LPM3770277 on CDK4, CDK6, Rb, P-Rb in HCC1806, and SUM159 cells at different times and doses.

Because we found that LPM3770277 was able to degrade CDK4/6 protein in two TNBC cell lines, we next examined the binding affinity, the time-dependent and dose-dependent effects of LPM3770277 on CDK4/6 protein. Binding experiment showed that LPM3770277 had a slightly weaker binding affinity on CDK4kinase, which may be due to the hydrophobic tags on LPM3770277 which could increase its steric hindrance (Supplementary Figure S1). Figure 3 showed the time-dependent and dose-dependent effects of LPM3770277 on CDK4/6 degradation. Initially LPM3770277 increased the expression of CDK4/6 protein (compare control vs. 4 h) but then the CDK4/6 expression level steadily and time-dependently decreased, reaching the maximal degradation at 72 h. At the same time, the degradation effect was clearly dose-dependent, with the concentration of 2 μM showing consistently higher degrading capability than lower concentrations. These results showed that LPM3770277 could bind to CDK4 kinase and degrade CDK4/6 protein in a time-dependent and dose-dependent manner.

### LPM3770277 Degraded CDK4/6 Protein *via* Proteasome

**FIGURE 4:**
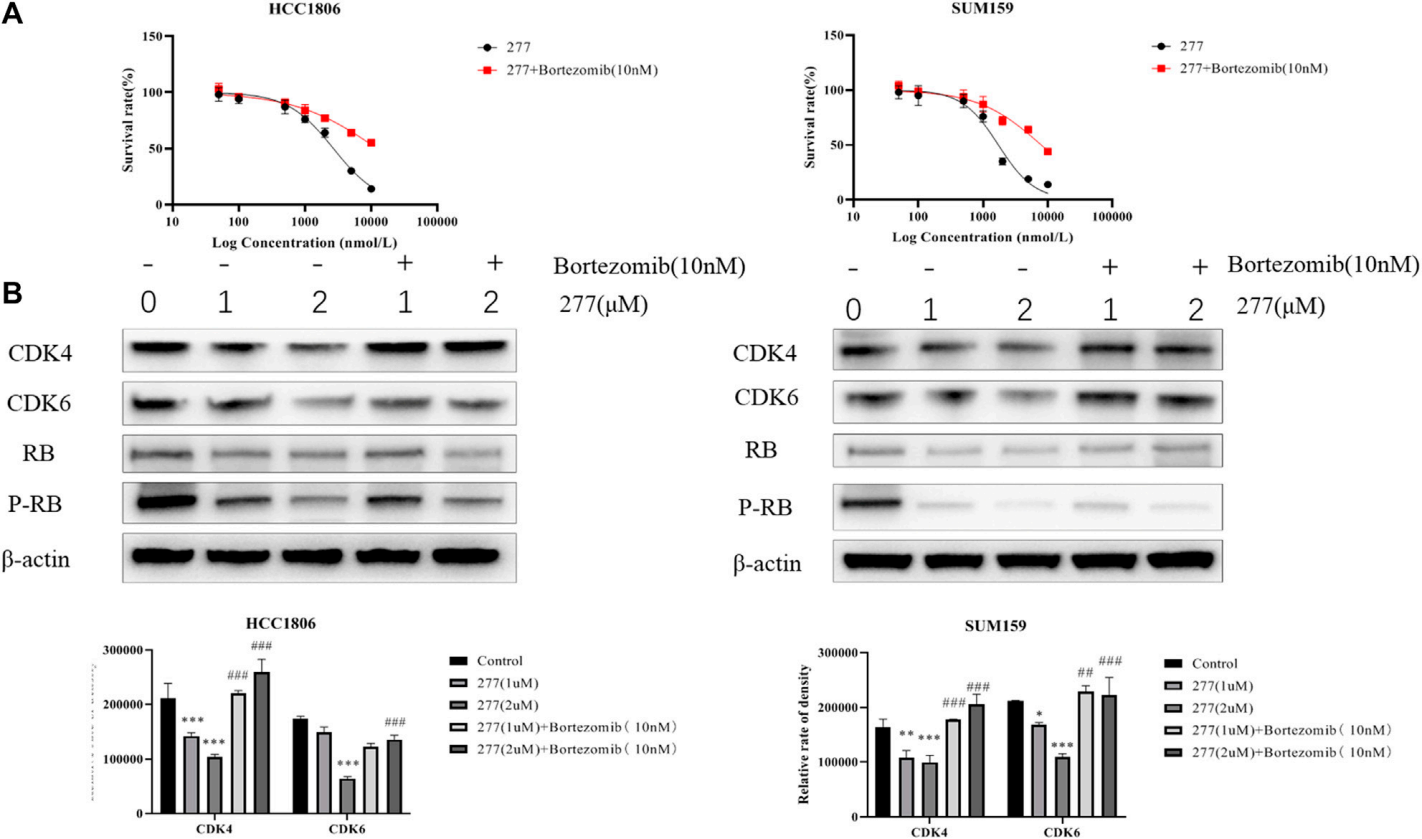
Effects of LPM3770277 on proteasome. Treatment48h. **(A)**: The effect of LPM3770277, the combined application of LPM3770277 and bortezomib on the survival rate of HCC1806 and SUM159 cells. **(B)**: The effect of LPM3770277, the combined application of LPM3770277 and bortezomib on the expression of CDK4, CDK6, Rb, P-Rb protein in two cell lines. *p < 0.05 vs. control, **p < 0.01 vs. control, ***p < 0.001 vs. control,# *p* < 0.05 vs. 277 (1 μM), ## *p* < 0.01 vs. 277 (1 μM), ## *p* < 0.001 vs. 277 (1 μM) Two-way ANOVA with a post-hoc Bonferroni test was used for all the statistical analyses.

Because proteasome is one of the major protein degradation machineries in eukaryotic cells, next we examined whether this mechanism also underlies the CDK4/6-degradating efficacy of LPM3770277. We used a proteasome inhibitor bortezomib to block proteasome activity and it was found that bortezomib (10 nM) significantly and almost completely prevented the CDK4/6-degradating activity of LPM3770277 (Figure 4). This result suggested that LPM3770277 degraded CDK4/6 protein *via* proteasome to exert its anti-cancer cell proliferation activity. In addition, we examined the effect of Abemaciclib on the degradation of LPM3770277 (Supplementary Figure S7). We added LPM3770277 for 4 h after administration of Abemaciclib and added Abemaciclib for 4 h after administration of LPM3770277, and then detected CDK4/6 protein expression. The results showed that when we added LPM3770277 after Abemaciclib for 4 h, CDK4/6 protein did not decrease; however, when exposed to LPM3770277 for 4 h, the degradation effect of Abemaciclib on CDK4/6 protein was attenuated. Combined with results from the target binding experiment, we believe this is due to the fact that the CDK4-binding ability of LPM3770277 was lower than that of Abemaciclib. Thus, when Abemaciclib first binds to CDK4/6, the binding sites become unavailable to LPM3770277 and LPM3770277 becomes ineffective. On the other hand, when LPM3770277 binds to CDK4/6 first, Abemaciclib could compete the binding sites with LPM3770277, thereby reducing its degradation effect.

### LPM3770277, Lysosome and Autophagy

**FIGURE 5:**
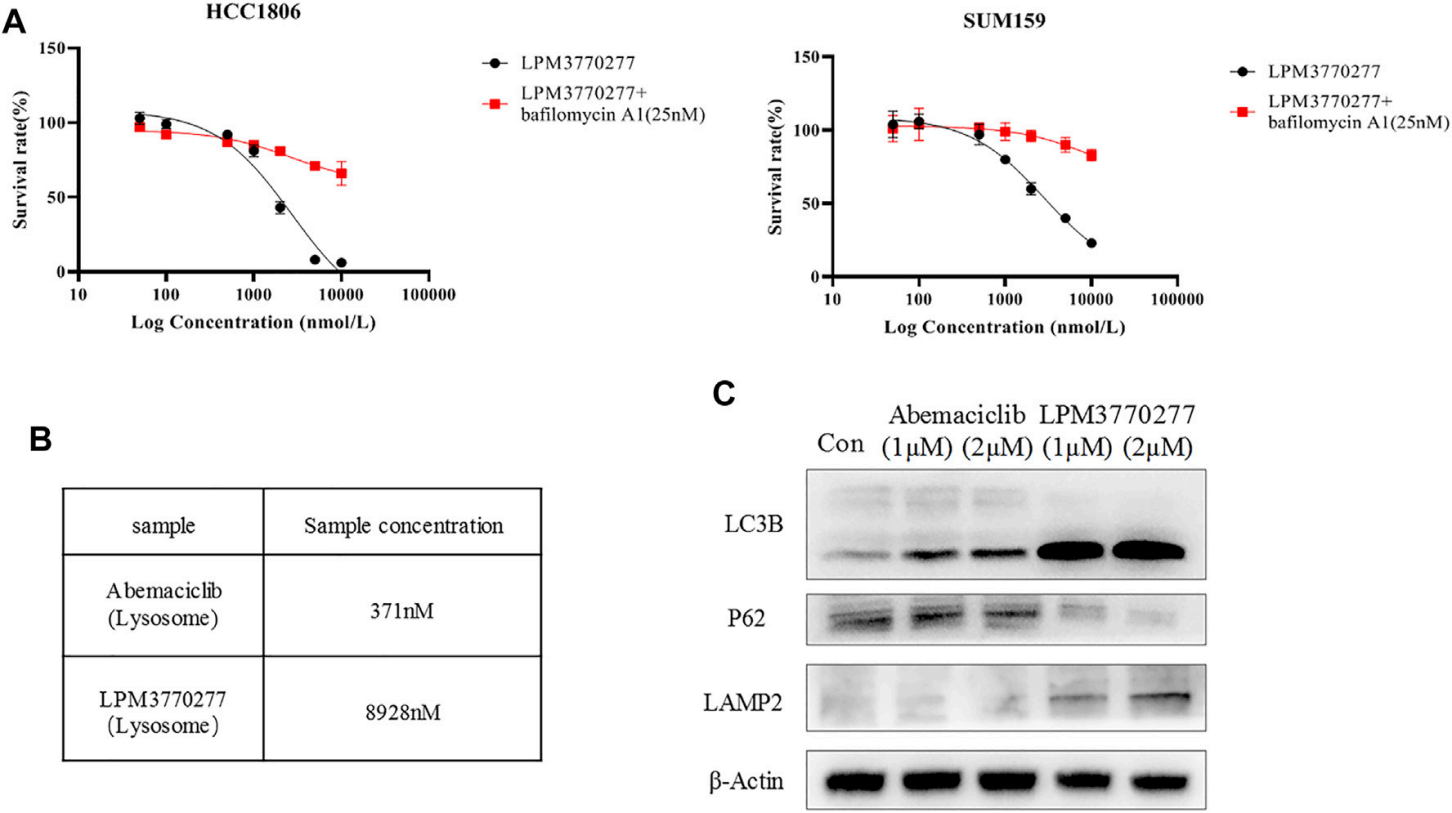
Effects of LPM3770277 on lysosome. **(A)**: The effect of LPM3770277, LPM3770277, LPM3770277 and lysosomal inhibitor Bafilomycin A1 (25 nM) in combination on HCC1806 and SUM159 cells. **(B)**: The concentration of the drug contained in the lysosome of HCC1806 cells. **(C)**: The effects of Abemaciclib and compound 277 on autophagy-related proteins LC3B, P62, LAMP2.

Besides proteasome, lysosome is another machinery that is involved in the degradation of many misfolded proteins. Next we tested the possibility that lysosome may also be involved in the efficacy of LPM3770277 to degrade CDK4/6 protein. We used a lysosomal inhibitor, bafilomycin A1, to study in combination with abemaciclib or LPM3770277. Surprisingly, bafilomycin A1 significantly enhanced the activity of abemaciclib. Further examination found that this is primarily due to the fact that the alkalescence on the abemaciclib parent structure can be isolated by lysosomes which prevented the compound to reach the target (Supplementary Figure S2A). What was equally surprising was that after inhibiting lysosomes by bafilomycin A1, the cytotoxic effect of LPM3770277 was significantly reduced (Figure 5A). The use of another lysosome inhibitor, NH4CL, led to similar results (Supplementary Figure S2B). To further solve this puzzle, we extracted the lysosomes of HCC1806 cells after drug treatment, and used HPLC to detect the drug content in the lysosome. The result showed that under the same condition, the drug concentration of LPM3770277 in the lysosome was 24 times higher than that of abemaciclib (Figure 5B). This suggests that LPM3770277 might operate *via* certain lysosome-dependent functions such as autophagy. To test this hypothesis, we examined the effects of abemaciclib and LPM3770277 treatments on autophagy-related proteins in HCC1806 cells. Figure 5C showed that LPM3770277 treatment increased the expression of LC3B protein and decreased the expression of P62 protein, both of which are crucial players in autophagy. These results indicate that LPM3770277 could significantly increase the autophagy level in HCC1806 cells, and that LPM3770277 may degrade CDK4/6 protein through lysosomal-mediated autophagy.

### Effects of LPM3770277 on Cell Cycle

**FIGURE 6:**
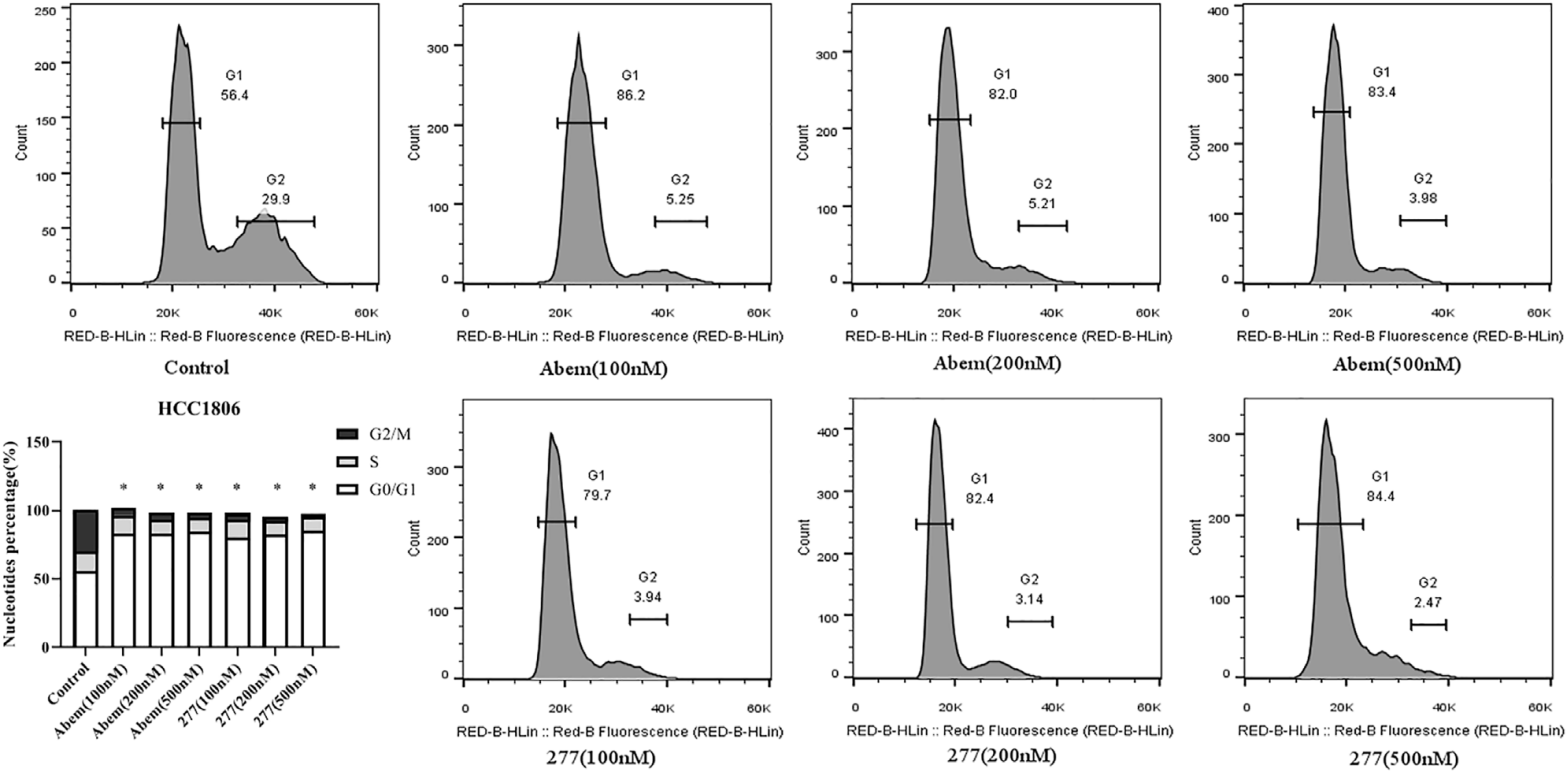
The effect of different concentrations of Abemaciclib and compound LPM3770277 on the cell cyle of HCC1806. *p < 0.05 vs. control Two-way ANOVA with a post-hoc Bonferroni test was used for all the statistical analyses. Abem: Abemaciclib, 277: LPM3770277.

We next examined the effects of LPM3770277 on cell cycle using flow cytometry. Results showed that LPM3770277 could significantly block HCC1806 and SUM159 cells in G1 phase (Figure 6; Supplementary Figure S3), which was similar to abemaciclib. This result suggested that the effect on cell arrest produced by the degradation of CDK4/6 protein by LPM3770277 is similar to direct inhibition of CDK4/6 by the CDK4/6 inhibitor abemaciclib.

### *In Vivo* Anti-Tumor Activity of Abemaciclib and LPM3770277 in Nude Mice

**FIGURE 7:**
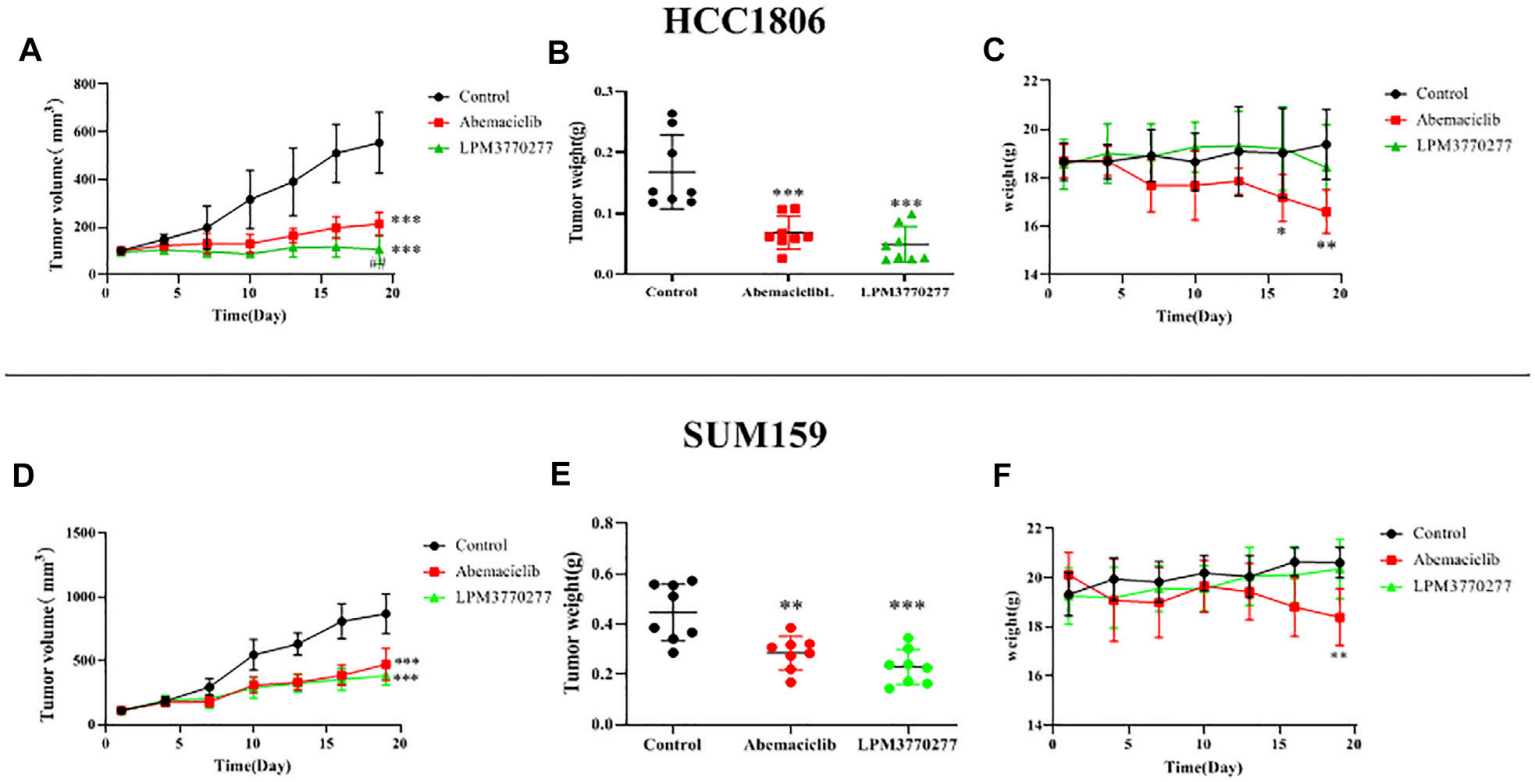
Results of xenograft tumor model of Abemaciclib and LPM3770277 in HCC1806 and SUM159 cells. **(A,D)**: Tumor volume. **(B,E)**: Tumor weight. **(C,F)**: Bodyweight were measured. *p < 0.01 vs. control, ***p < 0.001 vs. control, ## *p* < 0.01 vs. LPM3770277, Two-way ANOVA with a post-hoc Bonferroni test was used for all the statistical analyses.

Given the observed significant cytotoxic effects of LPM3770277 *in vitro*, the *in vivo* anti-tumor activity of LPM3770277 was evaluated in mouse HCC1806 and SUM159 cell xenograft tumor models. Before the *in vivo* experiment, the compound stability was assessed which found that LPM3770277 can reach an effective concentration in mouse liver microsomes (Supplementary Table S1). The *in vivo* results are shown in Figure 7 and Supplementary Figure S4. Although both abemaciclib and LPM3770277 can reduce tumor weight (Figures 7B,E) and tumor volume (Figures 7A,D), at longer timepoints LPM3770277 demonstrated better anti-tumor growth efficacy in both models (*p* < 0.05). It is noteworthy that abemaciclib (50 mg/kg) showed a trend of weight loss in the last few days of the experiment (Figure 7C) (*p* < 0.05), which was not observed in the LPM3770277 treatment group (Figure 7F). These results showed that LPM3770277 has higher anti-tumor efficacy and lower adverse effects in HCC1806 and SUM159 cell xenograft tumor models as compared to abemaciclib.

### *In Vivo* Degradation Effect of LPM3770277 on CDK4/6 Protein

**FIGURE 8:**
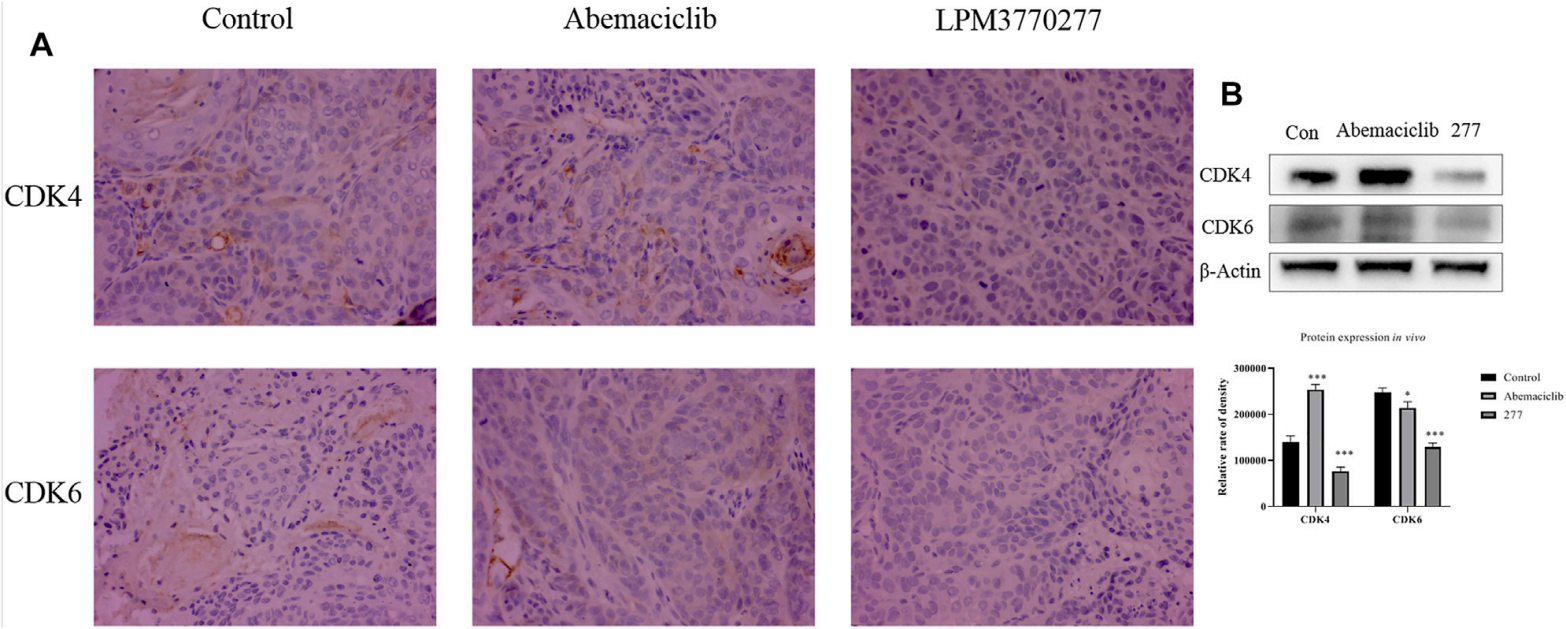
The effect of the compound on CDK4/6 protein in HCC1806 xenograft tumor. **(A)**: Immunohistochemical method test result. **(B)**: western bolt test result. *p < 0.01 vs. control Two-way ANOVA with a post-hoc Bonferroni test was used for all the statistical analyses. 277:LPM3770277.

In order to detect the expression of CDK4/6 protein in tumor tissue of nude mice, we processed tumor tissues from xenograft tumor models and detected the expression of CDK4/6 protein in tumors from different groups using immunohistochemistry (Figure 8A). Compared with the control group and the abemaciclib group, LPM3770277 treatment significantly reduced the expression of CDK4/6 protein in the tumor tissue. Similar result was also found in another xenograft tumor model (SUM159 cells) (Supplementary Figure S5A). The Western blotting results were consistent with this finding (Figure 8B; Supplementary Figure S5B).

### Potential Toxicity of LPM3770277 on Hematological System

**FIGURE 9:**
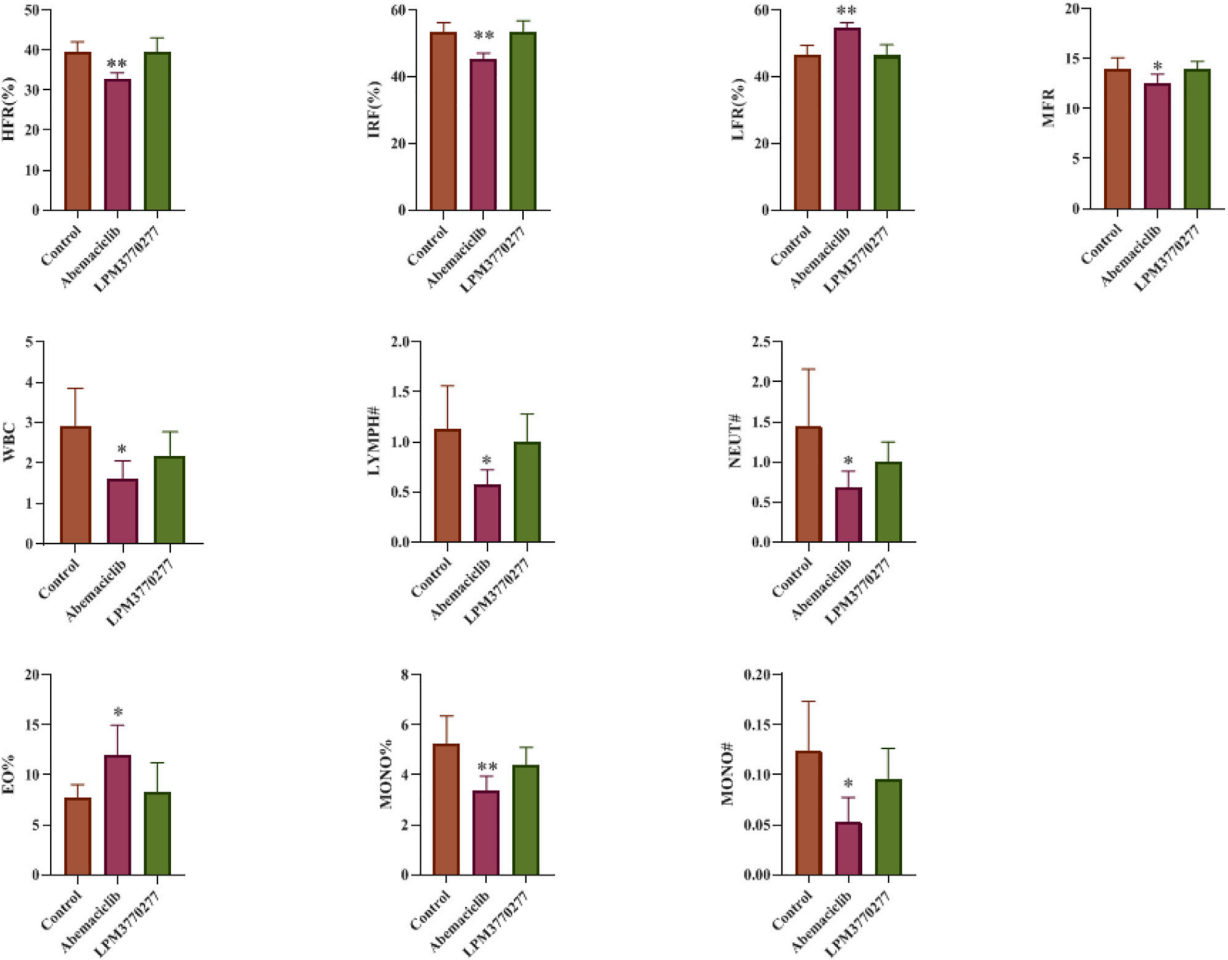
Effects of Abemaciclib and LPM3770277 on the blood of nude mice. HFR%, High fluorescence intensity reticulocyte percentage; IRF, Immature reticulocyte fraction; LFR%, Low fluorescence intensity reticulocyte percentage; MRF%, Medium fluorescence intensity reticulocyte percentage; WBC, White blood cell; LYMPH#, Lymphocyte-count; NEUT#, Neutrophil count; EO%, Percentage of basophils; MONO%, Percentage of Monocyte; MONO#, Monocyte count. *p ˂ 0.05 vs. control, **p ˂ 0.01 vs. control, One-way ANOVA with a post-hoc Bonferroni test was used for all the statistical analysis.

Because in the xenograft tumor models LPM3770277 showed no apparent effect on the body weight of nude mice, suggesting that LPM3770277 may have less severe adverse effects than abemaciclib, we extended the safety pharmacology test to the hematological system. Blood test results (Figure 9) showed that abemaciclib had a broad impact on numerous blood cells such as red blood cells (HFR, IRF, LFR, MFR) and white blood cells (WBC, LYMPH#, NEUT#, EO%, MONO#, and MONO%). Other blood cell indicators were largely normal (Supplementary Figure S6). In contrast, we observed no significant change on any of the indicators in LPM3770277-treated mice. These results indicate that LPM3770277 has little blood toxicity to nude mice.

## Discussion

The primary findings of the present study were that we developed and evaluated a novel CDK4/6 degrader, LPM3770277, based on hydrophobic tagging technology. LPM3770277 degraded CDK4/6 protein through the proteasome and autophagy pathways, and demonstrated significant cytotoxicity to Rb wild-type TNBC cells. In addition, we demonstrated that LPM3770277 had significant anti-tumor efficacy *in vivo* in mouse xenograft tumor models, and its hematological toxicity was much lower than that of CDK4/6 inhibitor abemaciclib. Together, these results demonstrate for the first time that the hydrophobic tagging-based CDK4/6 degrader has significant anti-tumor activity against TNBC and could be a potentially useful treatment strategy TNBC.

Tight regulation of the cell proteome is essential for the perfect interaction of different proteins necessary for normal cell function, survival and proliferation. Part of this regulatory network is to control protein synthesis and degradation (Finley, 2009; Lindquist and Kelly, 2011). The lysosomal system and the proteasome pathway play a central role in protein homeostasis (Tang et al., 2010; Bustamante et al., 2018; Wu et al., 2018). Currently, pharmacotherapeutic strategies are primarily based on target occupancy where inhibitors or modulators bind to disease-related proteins with sustained period of time to achieve clinical benefit (Adjei, 2006). However, this often leads to non-target constraints and side effects. Another option is an event-driven strategy, where the drug-target engagement triggers an event to modify disease-related proteins (Cromm and Crews, 2017). So far, such a strategy has only achieved limited success. Among them, nucleotide-based methods, such as small interfering RNA (siRNA), antisense oligonucleotides, or genome editing strategies, such as CRISPR-Cas9, only has limited use due to their low *in vivo* stability and narrow bioavailability (Whitehead et al., 2009; Kim et al., 2017; Lai and Crews, 2017). In addition, small molecules begin to be used to selectively induce the degradation of a variety of disease-relevant proteins, including proteolytic targeting chimeras (PROTACs), and hydrophobic tags (Wang et al., 2020). This study is an attempt to develop anti-TNBC cancer compounds using hydrophobic tags. We chemically modified the major metabolite of CDK4/6 inhibitor abemaciclib, M2, and developed three compounds and evaluated their *in vitro* CDK4/6-degradating activity, among which we identified LPM3770277 as the most promising candidate as it demonstrated significant CDK4/6-degradating efficacy. Through examining the time-dependent and dose-dependent relationships, we found that LPM3770277 exerted a long-lasting CDK4/6-degradating activity *in vitro*.

The main driving force for the correct folding of proteins is to minimize the number of hydrophobic groups exposed to water (Dill and MacCallum, 2012). When protein misfolding occurs, cellular quality control mechanisms are activated which lead to protein degradation through two major mechanisms: proteasome and lysosome (Kim et al., 2013). In an effort to decipher the CDK4/6-degradating mechanisms of LPM3770277, we utilized pharmacological inhibitors of proteasome and lysosome which confirmed that LPM3770277-induced CDK4/6 protein degradation was likely mediated through both machineries. In addition, biochemical characterization revealed that LPM3770277 likely induced cytotoxicity by promoting cancer cell autophagy. These mechanisms are drastically different from the clinically approved anticancer agent abemaciclib, which is a direct CKD4/6 protein inhibitor and does not involve protein degradation. Importantly, we found that both CDK4/6 protein degradation *via* LPM3770277 and CDK4/6 protein inhibition *via* abemaciclib lead to similar cell cycle arrest at G1 phase in TNBC cancer cells. This suggests that CDK4/6 degrader is a viable pharmacotherapeutic approach against TNBC, which may have similar or even better therapeutic efficacy to treat TNBC.

Safety is an important factor in the development of anticancer drugs. During the *in vivo* anti-cancer experiment, we noticed the interesting divergence between the CDK4/6 inhibitor and anticancer drug abemaciclib and LPM3770277 wherein abemaciclib produced a progressive body weight loss in mice but LPM3770277 had no apparent impact on the animals’ body weight. To expand the safety pharmacology testing with LPM3770277, we examined the effects of both compounds on hematological indicators in mice. Abemaciclib produced a broad hematological aberrancy in many blood cell-related indicators while LPM3770200 did not have any observable impact on the same blood cell indicators. This is particularly prominent and clinically relevant given that one major adverse effect of CDK4/6 inhibitors such as abemaciclib is lymphopenia syndrome. These results suggest that hydrophobic tags-based protein degraders could be a viable therapeutic strategy against cancer in general, and TNBC in particular, by providing comparable if not better therapeutic efficacy and preferable safety profile.

In summary, this study for the first time developed and characterized a novel CDK4/6 degrader, LPM3770277, based on hydrophobic tagging technology against TNBC. LPM3770277 was able to bind to and degrade CDK4/6 protein through proteasome and lysosome-induced autophagy mechanisms. In *in vivo* studies LPM3770277 demonstrated significant anti-TNBC cancer efficacy and superior safety as compared to the clinically used CDK4/6 inhibitor abemaciclib. Together, these results strongly support the validity of hydrophobic tagging in the development of protein-specific degraders against cancers such as TNBC. In addition, based on the preclinical efficacy and safety results, LPM3770277 seems to be a promising lead compound against TNBC that warrants further studies.

## Data Availability

The original contributions presented in the study are included in the article/Supplementary Material, further inquiries can be directed to the corresponding authors.
