# Supplementary figures and images for: LPM3770277, a Potent Novel CDK4/6 Degrader, Exerts Antitumor Effect Against Triple-Negative Breast Cancer
Source: Front Pharmacol. 2022 Apr 11;13:853993. doi: 10.3389/fphar.2022.853993 (PMC9037595; doi:10.3389/fphar.2022.853993)

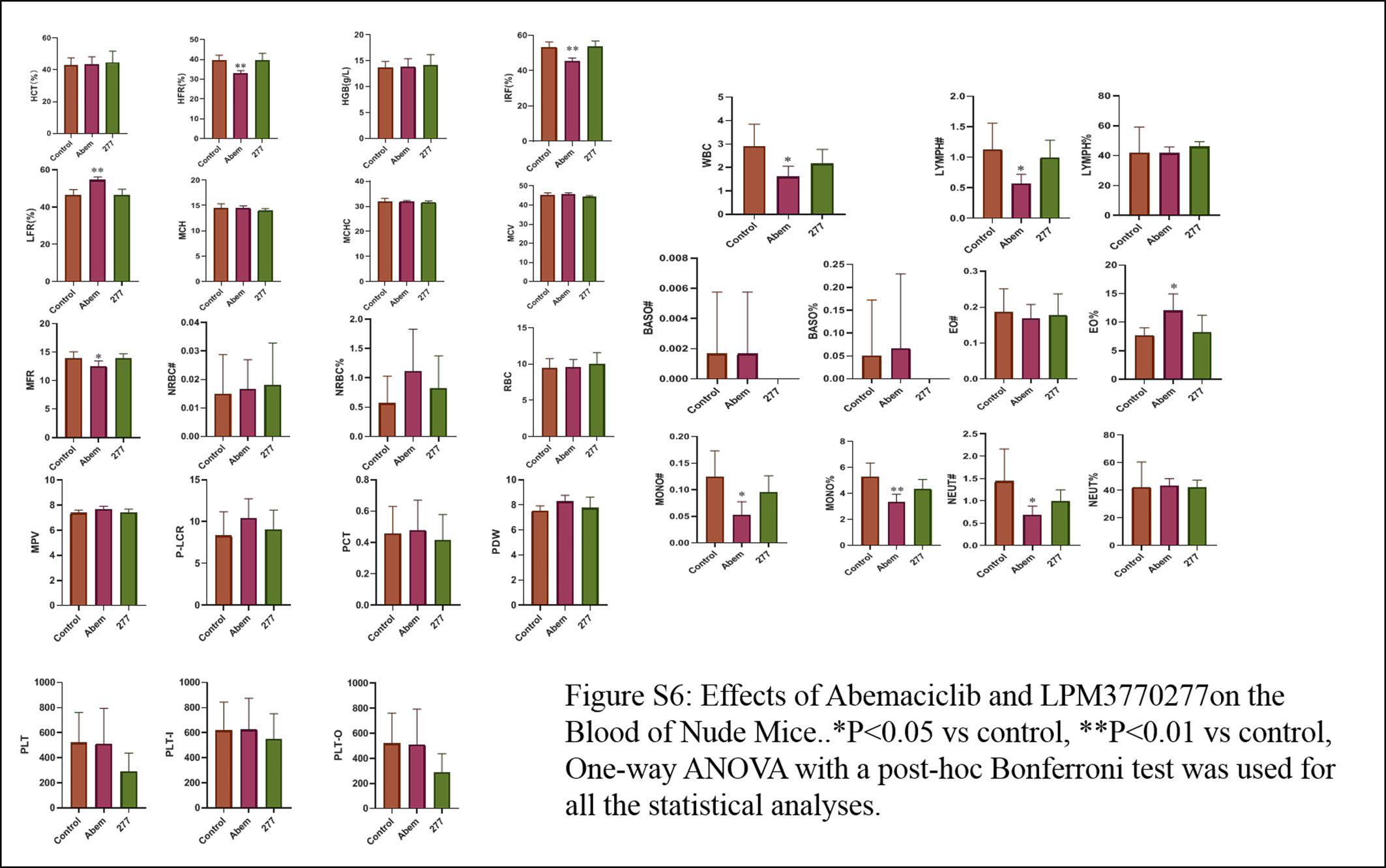

Supplement: Supplementary file 1 [file Image6.tif]

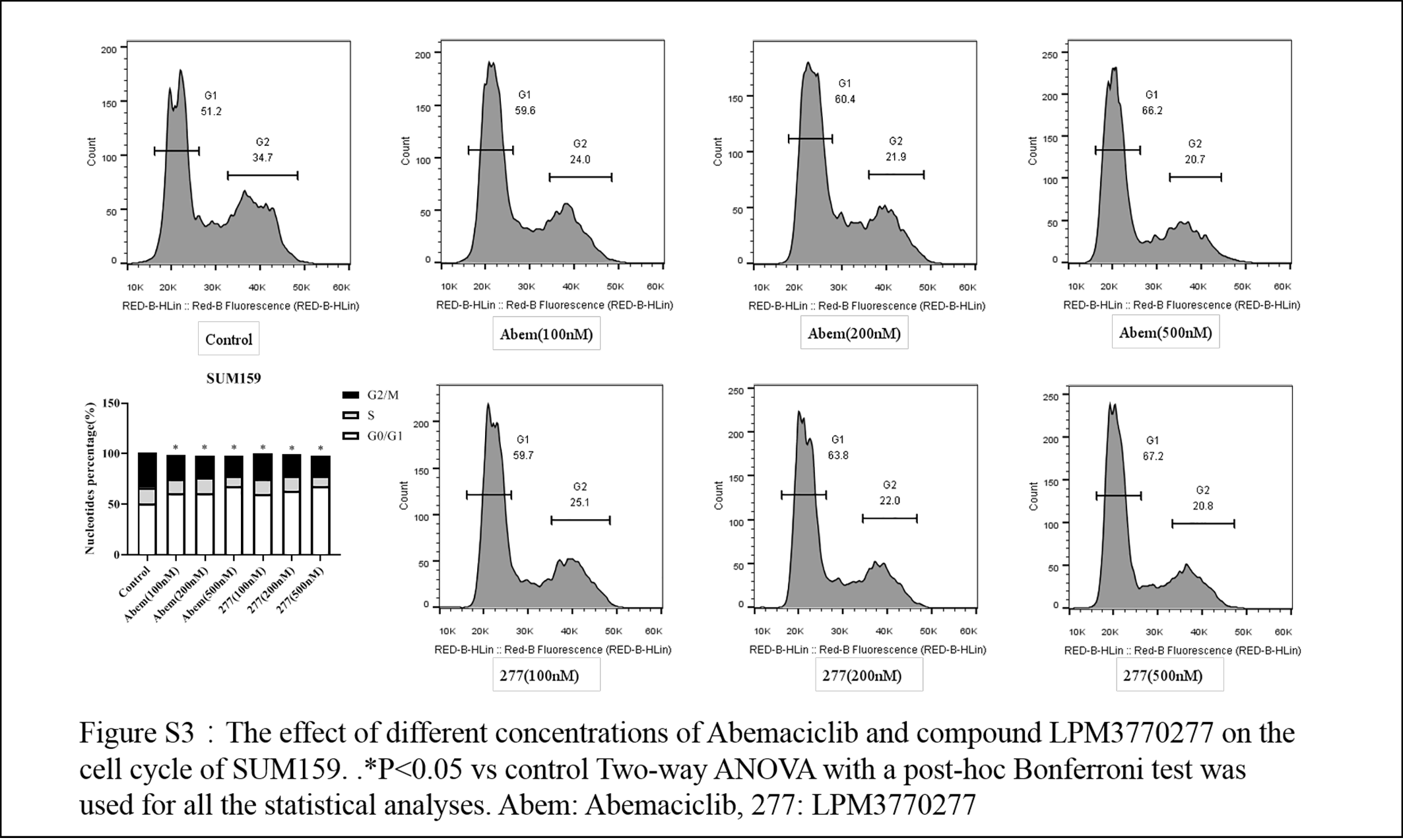

Supplement: Supplementary file 3 [file Image3.tif]

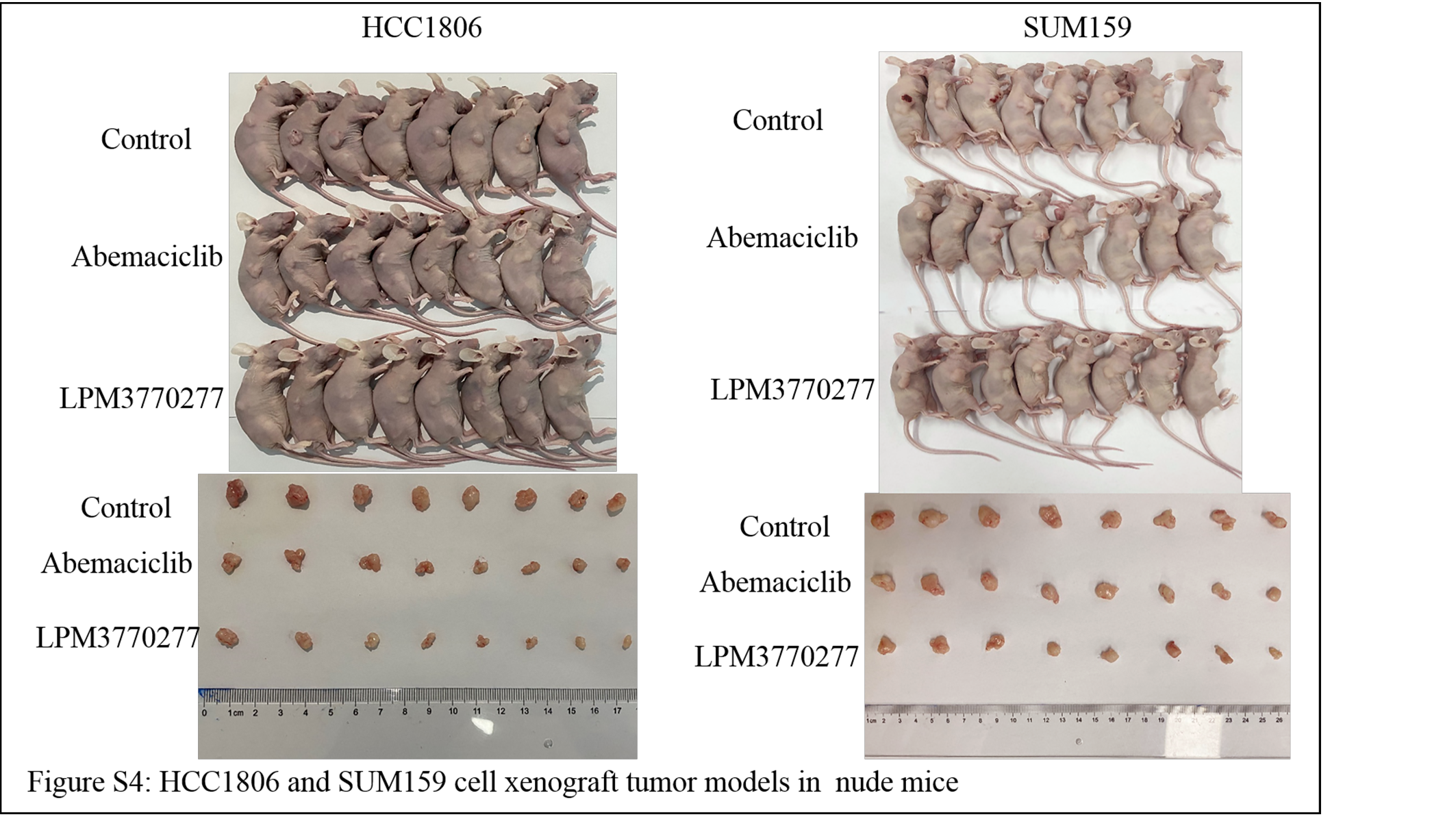

Supplement: Supplementary file 4 [file Image4.tif]

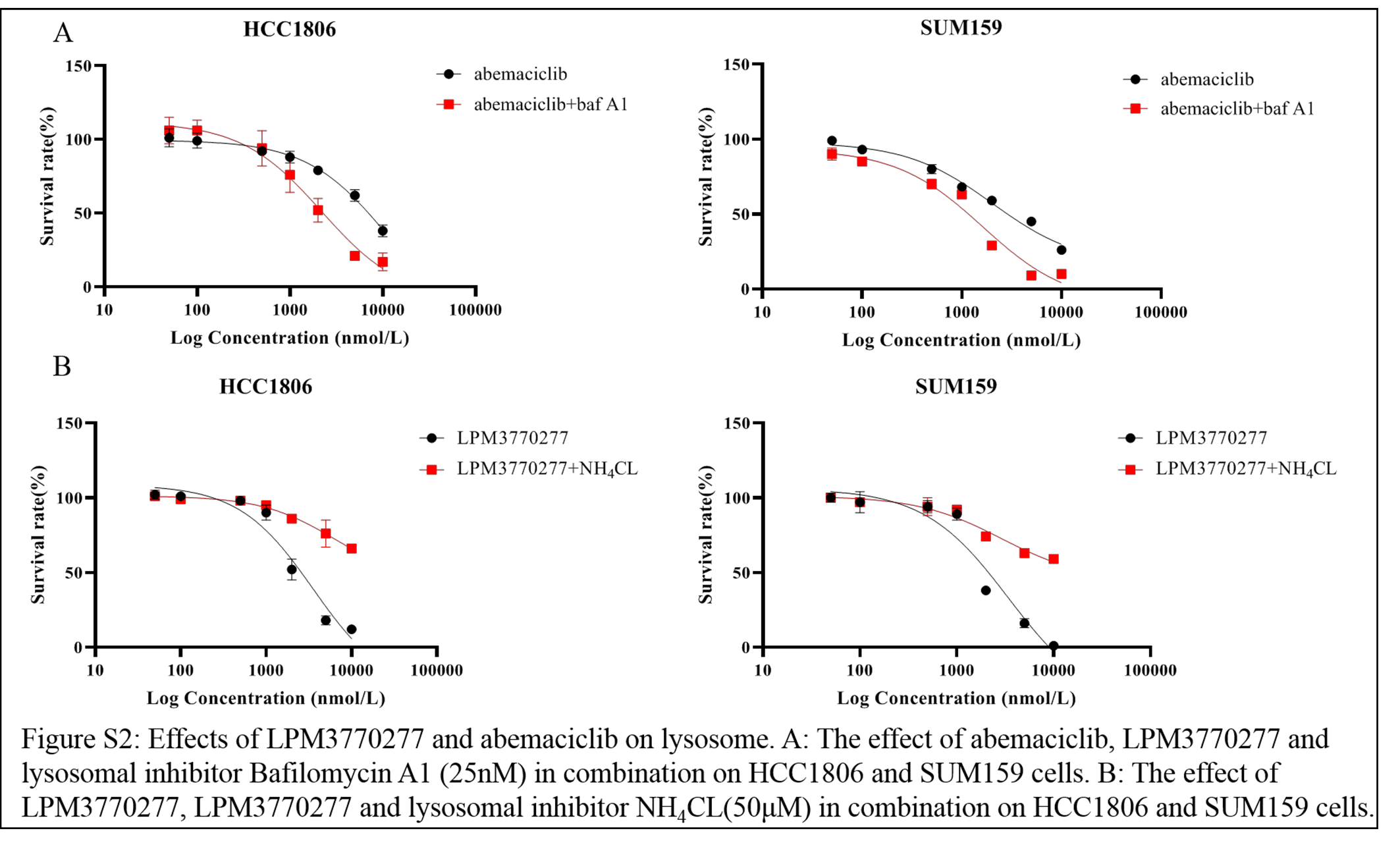

Supplement: Supplementary file 6 [file Image2.tif]

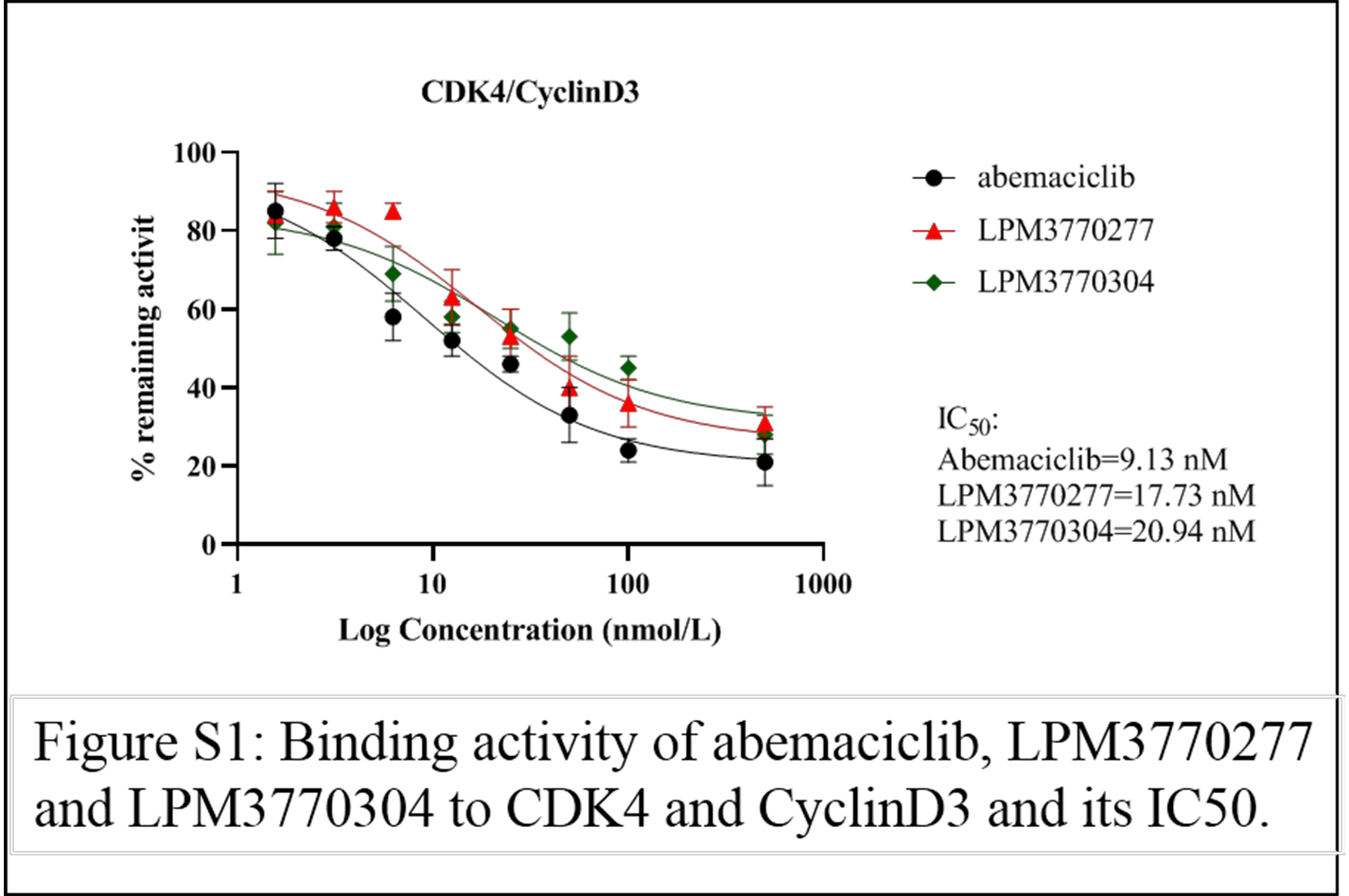

Supplement: Supplementary file 7 [file Image1.tif]

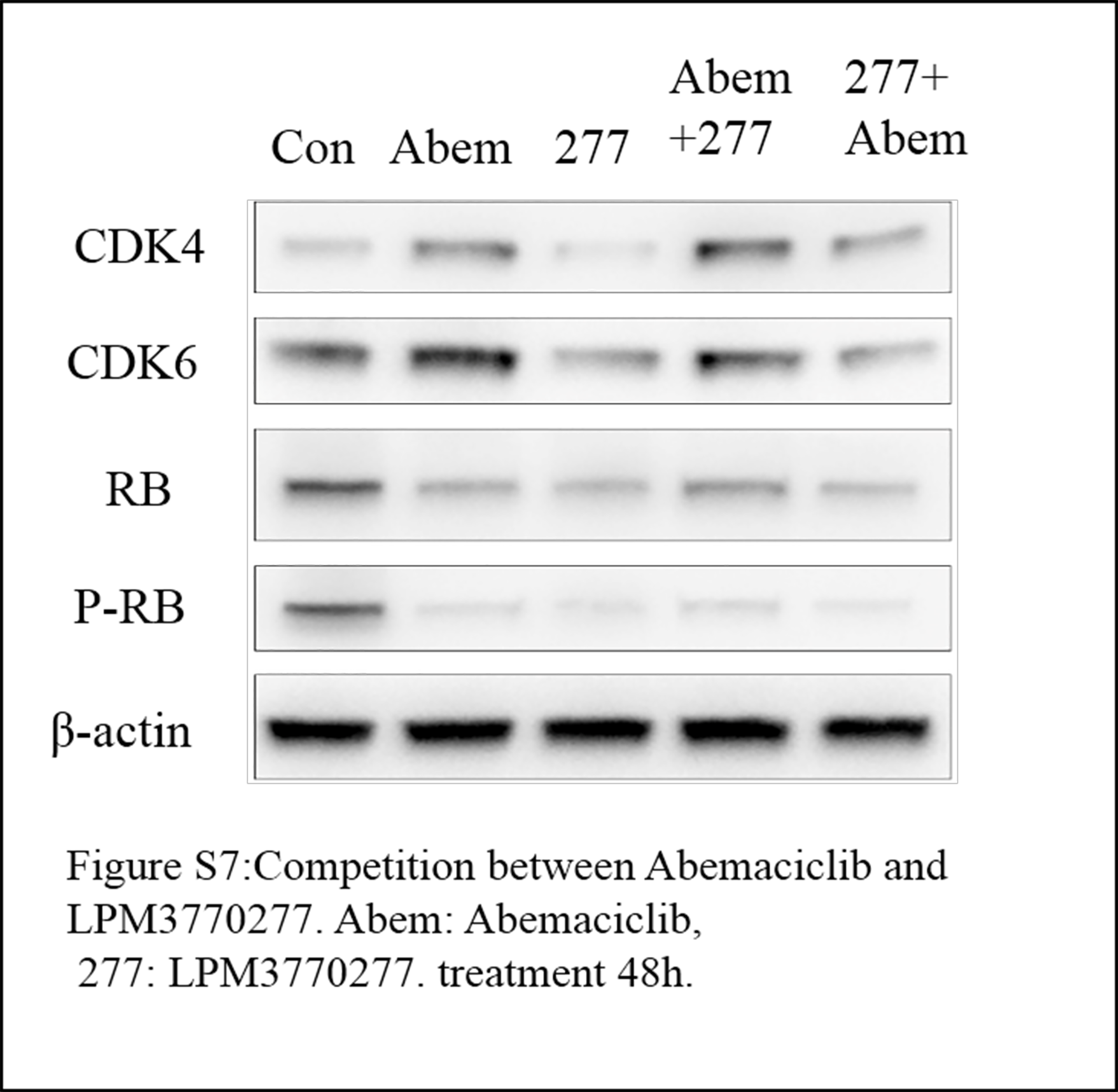

Supplement: Supplementary file 8 [file Image7.tif]

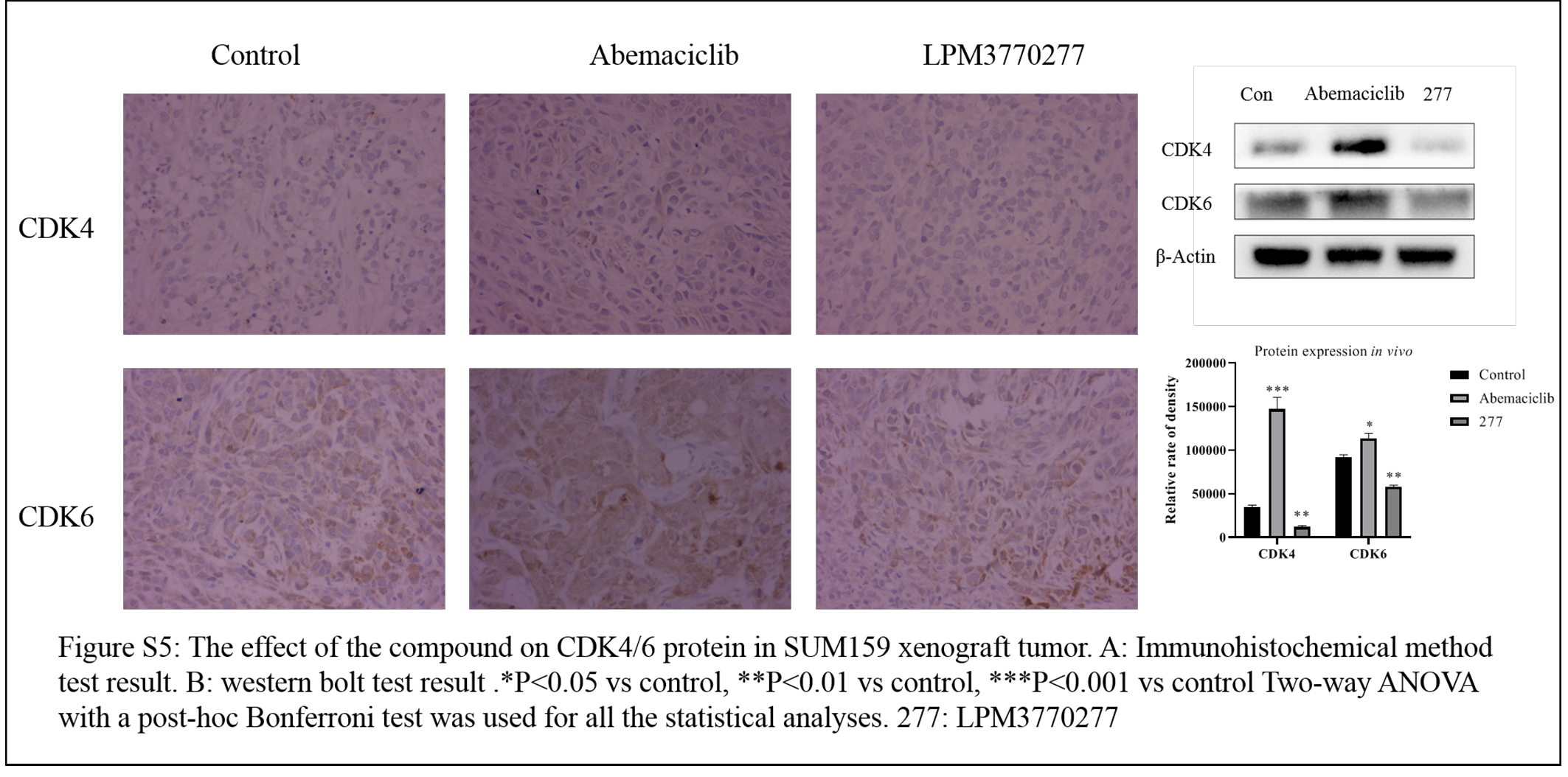

Supplement: Supplementary file 9 [file Image5.tif]
