# Supplementary material for: LPM3770277, a Potent Novel CDK4/6 Degrader, Exerts Antitumor Effect Against Triple-Negative Breast Cancer
Source: Front Pharmacol. 2022 Apr 11;13:853993. doi: 10.3389/fphar.2022.853993 (PMC9037595; doi:10.3389/fphar.2022.853993)
Supplement: Supplementary file 2 [file Table1.pdf]

compounds	species	Remaining amount after 60min incubation (%)	k (min^{-1})	$T_{1/2}$ (min)	$Cl_{int, microsome}$ ($\mu L \cdot min^{-1} \cdot mg^{-1}$ proteins)	$CL_{int, in vivo}$ (mL/min/kg)	Clearance Classification
LPM3770277	Human	61	0.0067	103	13.4	12.7	middle
	Rat	81	0.0041	169	8.20	14.8	Low
	Mouse	71	0.0051	136	10.2	41.3	middle

Table S1: Stability test of LPM3770277 in human, rat and mouse liver microsomes
