# Supplementary material for: LPM3770277, a Potent Novel CDK4/6 Degrader, Exerts Antitumor Effect Against Triple-Negative Breast Cancer
Source: Front Pharmacol. 2022 Apr 11;13:853993. doi: 10.3389/fphar.2022.853993 (PMC9037595; doi:10.3389/fphar.2022.853993)
Supplement: Supplementary file 5 [file Table2.pdf]

Table S2 Pharmacokinetic parameters

PK parameters	$AUC_{0-\infty}$ (h*ng/ml)	C_{max}(ng/mL)	$T_{1/2}$(h)	MRT(h)
Plasma	841	528	3.05	1.18
Tumor	388	163	-	1.75

Note: Three BABL/C mice in each group were orally administered with 57.9 mg/kg of LPM3770277 suspension. blood samples were collected at pre-dose (0 hr), and at post-dose 15 min, 1 hr, 2 hrs, 4 hrs, 8 hrs, 12 hrs, 24 hrs.
